# Bilateral Central Retinal Vein Occlusion as a First Presentation of Multiple Myeloma: A Case Report

**DOI:** 10.5811/cpcem.2022.4.55710

**Published:** 2022-07-27

**Authors:** Michael Andrew Tandlich, Kelly Williamson

**Affiliations:** * Northwestern University, Department of Emergency Medicine, Chicago, Illinois; † Northwestern University, Feinberg School of Medicine, Department of Emergency Medicine, Chicago, Illinois

**Keywords:** case report, bilateral central retinal vein occlusion

## Abstract

**Introduction:**

Acute presentation of multiple myeloma in the emergency department (ED) is an uncommon yet life-threatening clinical entity.

**Case Report:**

A 42-year-old male presented to the ED with severe generalized fatigue and vision changes most notable in his left eye. Bilateral central retinal vein occlusion (CRVO) was diagnosed on dilated fundus exam in the ED.

**Conclusion:**

The most common cause of CRVO in adults over age 50 is vascular disease, but in younger adults, conditions of systemic inflammation or hyperviscosity must be considered. Diagnosis of CRVO requires emergent ophthalmology consultation and further treatment with phototherapy, steroids, and potentially anti-vascular endothelial growth factor. Ultimately, patients require hematology/oncology and ongoing management of acute hyperviscosity syndrome. We present this case to increase awareness surrounding this diagnosis among emergency physicians. Multiple myeloma should be considered in young patients who present to the ED with bilateral CRVO, acute renal failure, and symptomatic anemia.

## INTRODUCTION

Multiple myeloma is a neoplasm characterized by the proliferation of plasma cells producing monoclonal immunoglobulins. While the presentation is often subacute or chronic, a small proportion of patients will present acutely. Patients may present with bone pain secondary to lytic lesions, an increased serum protein concentration, unexplained anemia, hypercalcemia, or acute renal failure. Although less common, acute presentations of multiple myeloma may present to the emergency department (ED), where it is imperative the emergency physician appropriately diagnose and manage this neoplasm. We report a case of bilateral central retinal vein occlusion (CRVO), acute renal failure, and symptomatic anemia in a man previously undiagnosed with multiple myeloma.

## CASE REPORT

A 42-year-old male presented to the ED, referred by his primary care physician for concern of anemia and vision changes. Over the previous two weeks, blurred vision to the left eye progressed to solid black centrally with sparing of the peripheral vision. Over the previous five days he also noticed right eye blurred vision. On review of systems, the patient endorsed experiencing occasional floaters and flashes bilaterally and unintentional weight loss of more than 60 pounds in the previous four months. He denied eye pain, headache, jaw claudication, scalp tenderness, fevers, or chills. Past medical history included only hypertension and hyperlipidemia, maintained on nifedipine 30 milligrams (mg) and simvastatin 20 mg daily, as well as strabismus of the left eye. The patient had been recently admitted to the hospital for symptomatic anemia, was found to have low vitamin B12 and borderline iron levels, had received a transfusion, and was started on ferrous gluconate and cyanocobalamin before being discharged. Social history included non-injection heroin use.

On the exam, he was well-appearing, resting comfortably in bed, in no distress. His vital signs were as follows: 36.6°C; blood pressure 160/87 millimeters of mercury (mm Hg); heart rate 75 beats per minute; respiratory rate 18 breaths per minute; and pulse oximetry 98% on room air. His head, ear, eye, nose and throat exam was notable for mild strabismus of the left eye compared with the right, as well as decreased central vision in the left eye. Fundoscopic exam was notable for significant retinal hemorrhage of the left eye. The remainder of the physical exam was unremarkable.

The blood work was significant for a red blood cell count of 2.31 million/microliter (M/μL) (reference range 4.30–5.80 M/μL); hemoglobin of 7.0 grams/deciliter (g/dL) (13–17.5 g/dL); mean corpuscular volume of 104 femtoliters (80–99 femtoliters), platelets of 94/ *μL* (140–390/μL), creatinine of 1.37 mg/ dL (0.6–1.3 mg/dL); glucose of 156 mg/dL (65–100 mg/dL); prothrombin time of 17.2 seconds (10.5–13.5 seconds); partial thromboplastin time of 28.7 seconds (24.8–38.4 seconds). Complete blood count differential was notable for absolute plasma cells of 0.1 K/uL, or 3% (0 K/uL), and red blood cell count morphology was notable for rouleaux. Calcium level was 8.9 mg/dL (8.3–10.5 mg/dL).

Given the patient’s vision change and findings of retinal hemorrhage noted on ED fundoscopic exam, ophthalmology was consulted. Their exam was notable for visual acuity: 20/60 without correction and 20/40 pinhole on right eye. On the left eye, he was able to count fingers only. His visual fields were full on right eye, full and central scotoma on left eye. Extraocular movements were intact bilaterally. and pupils were equal, round, and reactive bilaterally. Intraocular pressures were 9 mm Hg on the right eye and 7 mm Hg on the left eye (10–21 mm Hg). His slit lamp exam of the eyes bilaterally was notable for clear corneas, absence of conjunctival injection, round and flat irises, and clear lens and vitreous. His dilated fundus exam with 1% tropicamide and 2.5% phenylephrine revealed optic discs with sharp margins, pink rims, and no edema. The macula exam demonstrated possible edema on right and macular edema on left. The vessel exam was significant for diffuse hemorrhages throughout with vascular tortuosity of most vasculature and notable flame hemorrhage inferior to disc in both eyes.

The patient was diagnosed with bilateral central retinal vein occlusions, acute kidney injury, and symptomatic anemia. He received two liters of fluid. Consultation was made to hematology/oncology for presumed diagnosis of multiple myeloma. Due to the absence of other systemic signs or symptoms of hyperviscosity, the decision was made not to start plasmapheresis. Inpatient bone marrow biopsy confirmed the diagnosis of multiple myeloma, and the patient was started on steroids. On follow-up ophthalmology exams, the patient’s vision has maintained at 20/200 bilaterally. He continued to follow up in the hematology/ oncology clinic. He was initially started on bortezomib and methylprednisolone and then daratumumab and hyaluronidase human-fihj were added. At the time of manuscript submission, the patient was a candidate for bone marrow transplant and undergoing workup.

**CPC-EM Capsule**

What do we already know about this clinical entity?

*The appropriate diagnosis of an acute presentation of multiple myeloma is important, as it may result in life-threatening illness and severe morbidity if untreated*.

What makes this presentation of disease reportable?

*We present one of the first case reports of a patient with new onset multiple myeloma presenting to the Emergency Department with bilateral central retinal vein occlusion*.

What is the major learning point?

*The most common cause of central retinal vein occlusion (CRVO)in adults is vascular disease, but in younger adults, conditions of systemic inflammation or hyperviscosity must be considered*.

How might this improve emergency medicine practice?

*Multiple myeloma should be considered in young patients who present to the emergency department with bilateral CRVO*.

## DISCUSSION

To the best of our knowledge, we present the first case in the ED literature of a patient diagnosed with bilateral CRVO as the presenting symptom of multiple myeloma. Central retinal vein occlusion is commonly associated with systemic vascular disease, hypertension and diabetes, and most commonly presents in older patients. In patients younger than 50, CRVO is uncommon, and bilateral presentation warrants investigation into conditions that cause a hyperviscous or inflammatory state.^1^,^2^ Bilateral CRVO in particular is an extremely rare complication of an underlying hyperviscosity state, in which increased serum viscosity can result in vascular obstruction and tissue hypoxia. The hyperviscosity state in multiple myeloma is caused by excessive amounts of circulating immunoglobulins.^3^ In the case of multiple myeloma, CRVO is characterized by diffuse retinal hemorrhage and is associated with abnormalities in the retinal vein, such as engorgement, as well as optic disc swelling and macular edema.^4^ Symptoms of CRVO generally include painless changes in visual acuity, which may be sudden or progressive in onset.^5^ Additional symptoms associated with hyperviscous states include dyspnea, high output cardiac failure, and even myocardial infarction.

Bilateral CRVO is exceedingly rare, and its presentation has been documented in only a handful of case studies in association with hypercoagulable and hyperviscous states.^6^–^8^ In the rare case of bilateral CRVO, the differential diagnosis should include systemic lupus erythematosus, acute myeloid leukemia, Waldenstrom’s macroglobinemia, antiphospholipid antibody syndrome, and dysproteinemias.^9^ Multiple myeloma is most commonly diagnosed in the presence of hypercalcemia, renal insufficiency, anemia, and the presence of osteolytic bone lesions on skeletal radiography. However, definitive diagnosis requires bone marrow biopsy.^10^

Management involves an emergent consult to ophthalmology. Several therapies are available for the treatment of CRVO such as anticoagulants, photocoagulation, corticosteroids, and intravitreal injections, and therapy is often guided by an ophthalmologist.^11^ Further management of the underlying risk factors or etiology of the CRVO is also important and may require emergency physician referral to specialist care. When a hyperviscous state is deemed to be the etiology, treatment must be considered with supportive therapy such as fluids (as dehydration may worsen hyperviscosity), plasma exchange or plasmapheresis, and chemotherapy.^12^ Ultimately, corticosteroids and anti-vascular endothelial growth factor therapy may improve the visual acuity in patients experiencing CRVO. Adjuvant therapy such as retinal photocoagulation and surgical options have limited supporting data.^11^

In the case described above, the patient presented with bilateral CRVO leading to a diagnosis of multiple myeloma, associated with severe symptomatic anemia and acute renal failure.^13^,^14^ Acute presentation of multiple myeloma, therefore, must be considered in patients presenting to the ED with bilateral CRVO.

## CONCLUSION

This is one of the first case reports to document a patient with new onset of multiple myeloma presenting to the ED with bilateral central retinal vein occlusion. The appropriate diagnosis of an acute presentation of multiple myeloma is important, as it may result in life-threatening illness and severe morbidity if left untreated. Multiple myeloma should be considered in young patients who present to the ED with bilateral CRVO. Management includes consultation with ophthalmology and to hematology/oncology, and treatment for hyperviscosity syndrome should be considered.
